# La rétinopathie ponctuée albescente

**DOI:** 10.11604/pamj.2016.25.39.10172

**Published:** 2016-09-28

**Authors:** Taoufiq Ramdani, Rachid Sekhsoukh

**Affiliations:** 1 Service d’Ophtalmologie, Centre Universitaire Mohammed VI, Oujda, Maroc

**Keywords:** Rétinopathie ponctuée albescente, rétinopathie pigmentaire, héméralopie, électrorétinogramme, Retinitis punctata albescens, pigmentary retinopathy, night blindness, electroretinogram

## Image en médecine

La rétinopathie ponctuée albescente est une forme rare de rétinopathie pigmentaire. La transmission est classiquement autosomique récessive. Il s’agit, dans la plupart des cas, d’une mutation dans le gène RLBP1. Elle présente les mêmes caractéristiques cliniques d'une rétinopathie pigmentaire classique et se caractérise par la présence de nombreuses taches blanches au niveau de l'épithélium pigmentaire parsemées au niveau de tout le champ rétinien. L'électrorétinogramme révèle par ailleurs une atteinte du système scotopique avec extinction globale dans les formes avancées. Actuellement, les recherches notamment le génie génétique, est en plein essor afin d’améliorer le pronostic visuel des patients atteints de ce syndrome. Nous rapportons le cas d’un enfant âgé de 16 ans dont des parents sont consanguins, qui a consulté pour héméralopie et chez qui l'examen ophtalmologique trouve aux deux yeux une acuité visuelle à 10/10 P2 avec un examen normal du segment antérieur. L’examen du fond d’œil trouve de nombreuses taches blanches disséminées. Les explorations électrophysiologiques ont permis de confirmer le diagnostic de rétinite ponctuée albescente.

**Figure 1:**
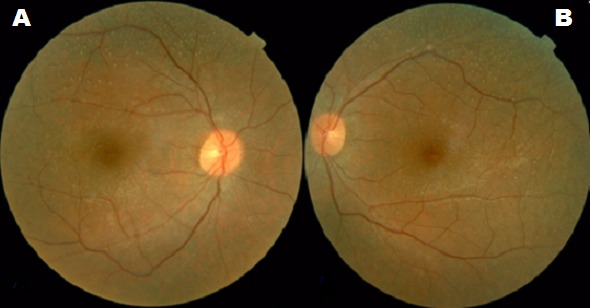
Rétinophtographies en couleur: A) œil droit; B) œil gauche: présence de nombreuses taches blanches au niveau de l’épithélium pigmentaire parsemées au niveau de tout le champ rétinien

